# Therapeutic antisense oligonucleotides against cancer: hurdling to the clinic

**DOI:** 10.3389/fchem.2014.00087

**Published:** 2014-10-14

**Authors:** Pedro M. D. Moreno, Ana P. Pêgo

**Affiliations:** ^1^Instituto de Engenharia Biomédica, Nanobiomaterials for Targeted Therapies GroupPorto, Portugal; ^2^Faculdade de Engenharia, Universidade do PortoPorto, Portugal; ^3^Instituto de Ciências Biomédicas Abel Salazar, Universidade do PortoPorto, Portugal

**Keywords:** antisense, oligonucleotides, cancer, therapeutics, nanomedicine

## Abstract

Under clinical development since the early 90's and with two successfully approved drugs (Fomivirsen and Mipomersen), oligonucleotide-based therapeutics has not yet delivered a clinical drug to the market in the cancer field. Whilst many pre-clinical data has been generated, a lack of understanding still exists on how to efficiently tackle all the different challenges presented for cancer targeting in a clinical setting. Namely, effective drug vectorization, careful choice of target gene or synergistic multi-gene targeting are surely decisive, while caution must be exerted to avoid potential toxic, often misleading off-target-effects. Here a brief overview will be given on the nucleic acid chemistry advances that established oligonucleotide technologies as a promising therapeutic alternative and ongoing cancer related clinical trials. Special attention will be given toward a perspective on the hurdles encountered specifically in the cancer field by this class of therapeutic oligonucleotides and a view on possible avenues for success is presented, with particular focus on the contribution from nanotechnology to the field.

## Opening the therapeutic landscape by evolution of nucleic acids chemistry

Oligonucleotides have been under investigation for over 30 years, whilst achieving only two approved drugs. Those were, Fomivirsen, approved by the FDA in 1998 for the treatment of cytomegalovirus retinitis in patients with AIDS, but discontinued for low demand, and Mipomersen, FDA approved in 2013, targeting ApoB100 for the treatment of homozygous familial hypercholesterolaemia (HoFH), a rare genetic disorder that leads to excessive levels of low-density lipoprotein (LDL) cholesterol. These are both single-stranded antisense oligonucleotide drugs (most commonly known as AONs) that together with siRNA (a double-stranded oligonucleotide) make up, at present, the therapeutic antisense oligonucleotide field. In this paper more emphasis will be put on AONs due to their longer time in development and history of clinical trials.

Progress in this field has been proceeding at a steady but somewhat slow pace, driven mostly by the speed at which the different intra and extracellular obstacles encountered by the oligonucleotide drugs are being tackled. The most important hurdles have been (i) the poor stability against extra- and intracellular degradation (mostly by action of nucleases), (ii) inefficient intracellular delivery to target cells or tissues, (iii) inadequate affinity toward the intended target sequence and (iv) potential off-target/toxicity effects. Finally for most applications (v) immunostimulation has also been a matter of concern.

The pursuit of clinically relevant antisense drugs has led the field to develop different types of chemical modifications to native DNA or RNA in an attempt to overcome the aforementioned limitations. Most widely used modifications can be divided in two simple categories: (a) backbone structure and (b) sugar ring modifications (Table 1).

**Table 1:**
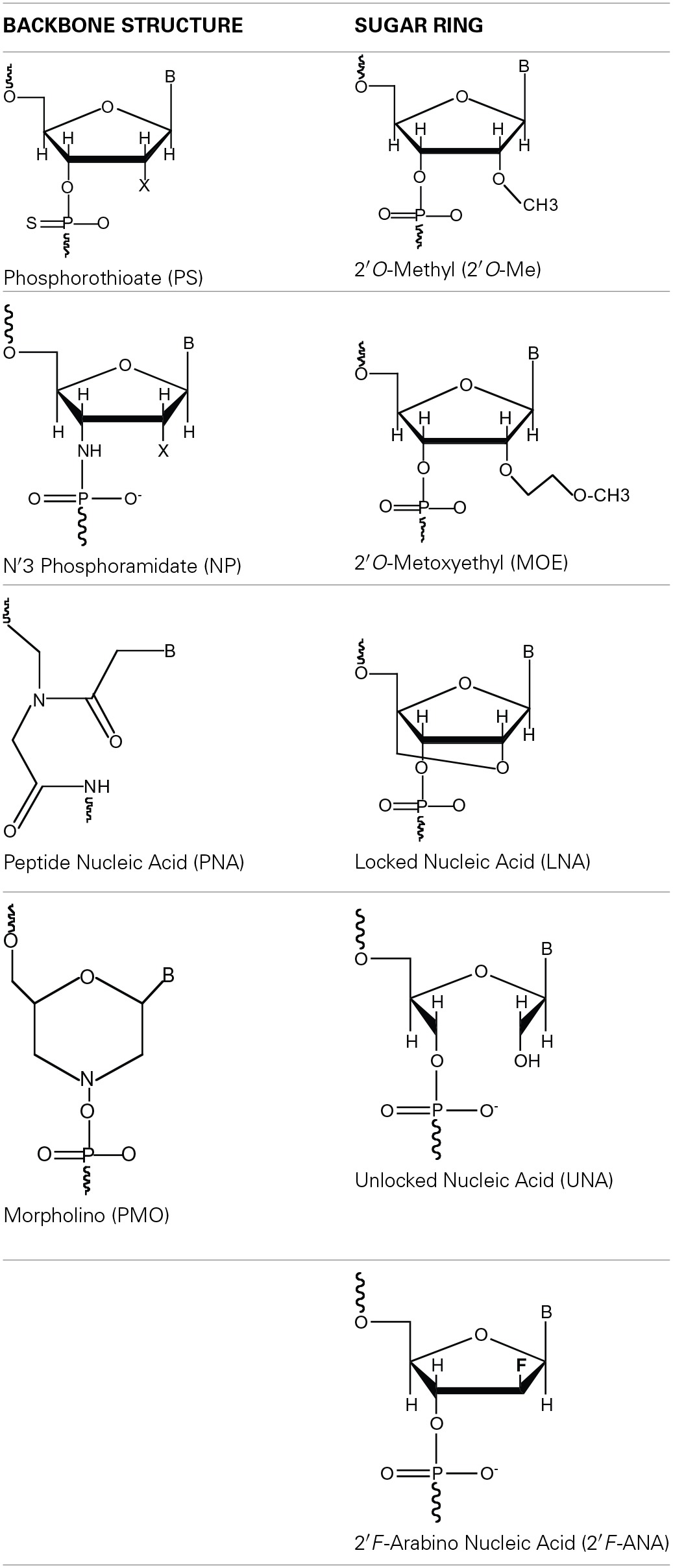
**Common nucleic acids modifications divided by category**.

The main goals of these chemical modifications have been to achieve increased resistance to degradation by exo- and endo-nucleases; increase affinity, and in some cases selectivity, toward target RNA/DNA sequences and to modulate the immunostimulation properties of the oligonucleotides.

In the case of phosphorothioate (PS) modification (one of the first and widely used modifications introduced in therapeutic antisense oligonucleotides) (Eckstein, 1967), it has also led to better pharmacokinetics and extended circulation times for AONs systemically applied in a “naked” form (i.e., unprotected by delivery agents). This effect has been attributed to unspecific binding to serum proteins such as albumin (Srinivasan et al., 1995; Crooke et al., 1996; Watanabe et al., 2006).

Mechanistically, AONs work via binding to a specific RNA target sequence resulting in the block of RNA function. This can be achieved through steric hindrance (non-degradative pathway) and concomitant RNA translation block, or target degradation. The latter occurs by the action of an endogenous enzyme, RNase H, or alternatively, by a catalytic cleavage activity embedded into the oligonucleotide itself (e.g., ribozymes and DNAzymes) (Bennett and Swayze, 2010). Notably, the non-degradative mechanism, through steric hindrance, has recently been exploited, with great success, for modulation of pre-mRNA splice patterns by affecting the binding of trans-splicing regulatory factors to the pre-mRNA (Hammond and Wood, 2011; Bestas et al., 2014; Disterer et al., 2014).

The mechanism of action of AONs has to be carefully considered when deciding for the type of nucleotide modifications and design of the oligonucleotide (here including number and position of modified nucleotides). Thus, in contrast to PS modifications that maintain the anionic character of oligonucleotides, PNAs and PMOs completely substitute the phosphodiester linkage by either a polyamide backbone (Nielsen et al., 1991) or a phosphorodiamidate group (Summerton and Weller, 1997), respectively, hence being uncharged nucleotide analogs. On the other hand sugar ring modifications can influence the nucleoside conformation promoting the preferential adoption of an A-form (dsRNA type) or B-form (dsDNA type) helix when in a double-stranded structure. In the case of 2′*O*-Me RNA (Kawai et al., 1992; Nishizaki et al., 1997), MOE (Manoharan, 1999) and LNA (Koshkin et al., 1998; Obika et al., 1998), all promote the A-form while 2′*F*-ANA (Berger et al., 1998) the B-form. Contrasting all the aforementioned, UNA, with its unlocked ring configuration, does not impart any conformation restrictions (Pasternak and Wengel, 2011).

All of the abovementioned nucleotide modifications have thus been used with few restrictions when designing steric hindrance AONs, since their incorporation mainly focus on achieving enhanced binding affinity and selectivity toward a target sequence. In contrast, the design of AONs for target degradation through the action of RNAse H has to obey the enzyme's structural preferences for cleaving DNA/RNA duplexes (Minshull and Hunt, 1986; Nakamura et al., 1991). Hence, all modifications too divergent from natural DNA nucleotides need to be carefully considered to not hinder the enzyme action. This can be accomplished by the design of “gapmer” AONs containing the modified nucleotides on the 5′ and 3′ terminus flanking a central unmodified DNA nucleotides stretch (Monia et al., 1993; Stanton et al., 2012). Specifically, in the case of the two approved drugs, Fomivirsen is a PS modified DNA oligonucleotide applied by intraocular injection, whereas Mipomersen is a second generation AON gapmer with MOE modifications at the ends and PS throughout, applied as an intravascular injection.

## Brief overview on antisense oligonucleotides clinical trials related to cancer

An increasing number of clinical trials with AONs are ongoing, which shows that the field is rapidly forwarding. In Table 2 a list of recently completed and on-going clinical trials is presented.

**Table 2:** **On-going and recently completed anti-cancer AON clinical trials**.

**DRUG**	**AON (carrier)**	**TARGET**	**INDICATION**	**STATUS**	**DEVELOPER**
Custirsen (OGX-011)	2′-*O*-MOE-PS gapmer ODN (“naked”)	Clusterin	(i) castrate resistant prostate cancer; (ii) non-small cell lung cancer	I and (ii) Phase III (recruiting)	OncoGenex
EGFR antisense DNA	Phoshorothioate ODN (“naked”)	EGFR	Advanced Head and Neck Squamous Cell Carcinoma	Phase I/II (recruiting)	University of Texas
Apatorsen (OGX-427)	2′-*O*-MOE-PS gapmer ODN (“naked”)	Hsp27	prostate cancer; pancreatic; non-squamous non-small cell lung cancer; other	Phase II (recruiting)	OncoGenex
ISIS-STAT3Rx (ISIS 481464/ AZD9150)	cEt-PS gapmer ODN (“naked”)	STAT3	Lymphoma; hepatocellular carcinoma	Phase I/II (recruiting)	Astrazeneca (ISIS Pharmaceuticals)
ISIS-ARRx (AZD5312)	cEt-PS gapmer ODN (“naked”)	Androgen Receptor	Advanced solid tumors (prostate cancer indications)	Phase I (recruiting)	Astrazeneca (ISIS Pharmaceuticals)
Trabedersen (AP 12009)	Phoshorothioate ODN (“naked”)	TGFβ 2	(i) Pancreatic Neoplasms, Melanoma, Colorectal Neoplasms; (ii) Glioblastoma; Anaplastic Astrocytoma	(i) Phase I; (ii) Phase IIb (both completed)	Isarna Therapeutics
EZN-2968	LNA-PS gapmer ODN (“naked”)	HIF-1α	Advanced solid tumors	Phase I (completed)	Enzon Pharmaceuticals (Santaris Pharma)
LErafAON-ETU	DNA-PS modified at 5′ and 3′ end (lipossome)	c-raf	Advanced Cancer	Phase I (completed)	INSYS Therapeutics Inc

*ODN—oligodeoxynucleotide; cEt—constrained Ethyl*.

Other studies have unfortunately failed, in different phases, to reach their expected endpoints or to show significant benefit, leading to a stop in the corresponding AON development. Some aspects of antisense technology have contributed to this and are next discussed.

## Challenges for antisense technology—1. unspecific modes of action

Along their development path, oligonucleotides have unraveled much of their potential but also many of their limitations.

As discussed above, introduction of PS modifications led to the first evidences that antisense drugs could become a reality in a clinical setting, essentially by increasing resistance to degradation and extending circulation times after systemic administration (mostly due to unspecific serum-protein binding). These properties improved the oligonucleotide therapeutic potential, despite some decreased affinity for the target sequence (when comparing to regular DNA oligonucleotides) (Kibler-Herzog et al., 1991). On the other hand, this unspecific protein binding feature can potentially lead to associated toxicities or cellular effects not entirely sequence specific, such as complement activation, increased coagulation times and unwanted immune activation (Brown et al., 1994; Krieg and Stein, 1995; Henry et al., 1999; Mou et al., 2001; Krieg et al., 2003; Senn et al., 2005). These effects, however, are most often oligonucleotide length and concentration dependent (Webb et al., 2001). Immune activation, on the other hand, is also enhanced by specific nucleotide sequences (CpG motifs) (Barchet et al., 2008), although this can be minimized by different types of nucleotide modifications (Henry et al., 2000). Nevertheless, immune activation is an important factor that has previously led to erroneous interpretations of data when inhibition of tumor growth was not primarily driven by the antisense mechanism but by the immunostimulatory properties of CpG sequences found in certain AONs (Badros et al., 2005; Gekeler et al., 2006). Regarding potential PS-derived unspecific cellular effects these have been proposed to affect the mechanism of action of an anti-cancer oligonucleotide drug by the down-regulation of several anti-apoptotic proteins and glycolytic enzymes. These were actually seen as important contributors to the apoptotic action (Stessl et al., 2009; Winkler et al., 2010).

Another important concern relates to hybridization dependent toxicity, deriving from exaggerated pharmacological action (a consequence also seen with any other chemical drug), or off-target hybridization. The latter can be minimized by designing the antisense drug taking into account a detailed bioinformatics analysis for identification of both, genes with perfect matches or with partial complementarity (looking out for 1–3 mismatches as the most relevant ones) (Bennett and Swayze, 2010).

The above considerations have raised some difficulties, especially *in vivo*, for the exact prediction of the mechanism of action of an antisense drug and are among the causes probably hampering a more resolute demonstration of the therapeutic relevance of antisense drugs toward not only cancer but also other diseases in general. This concern can be demonstrated by the case of the antisense drug LY2275796 (a second generation AON with PS and MOE modifications targeting eIF-4E) where, besides target gene downregulation, housekeeping genes were considerably affected as well, raising the question to whether the antisense action was sequence specific or also mediated by off-target effects (Hong et al., 2011).

This scenario only reinforces the need for an in-depth pharmacologic and pharmacokinetic analysis at the preclinical stage of AON development.

## Challenges for antisense technology—2. delivery

The efficient and targeted delivery of nucleic acid therapeutics is seen as, if not the biggest, one of the most important challenges for this class of drugs. The most commonly used nucleic acids drugs (namely, plasmid DNA, siRNA and AONs) have specific features influencing their cellular uptake and delivery vector development. AONs, due to the short chain size have very low charge density, in addition, being single-stranded, they have the aromatic bases exposed (not buried inside a double helix), which confers a slight hydrophobic character to the molecule. These properties, enable some level of interaction with the cell membrane, which can be further potentiated by PS modifications, making possible, although still extremely inefficient, their use without the help of any vector formulations (Watts and Corey, 2012). This has been recently emphasized by the demonstration that short LNA modified AONs were able to sustain gene downregulation in a large variety of cell lines when administered *in vitro* unassisted by transfection agents (also referred as gymnotic delivery), although some cell lines still seem to be completely refractory to this type of AON uptake (Stein et al., 2010). The results obtained by gymnotic delivery seem to correlate well with the obtained *in vivo* gene silencing efficiencies for the “naked” LNA administration; in fact, a better prediction of *in vivo* potency was obtained in comparison to data resulting from transfection-mediated *in vitro* AON delivery, a more standard method to preliminarily analyze AON efficiency (Stein et al., 2010). A similar study showed downregulation of different cancer gene targets, by the gymnotic delivery of LNA-AON in over 30 cell lines, although discrepancies between both studies are seen when relating intracellular localization of the AONs (nuclear vs. cytoplasmatic) and efficient down-regulation activity (Zhang et al., 2011).

Despite several studies demonstrating some activity when using “naked” AONs *in vivo*, and their wide tissue distribution, it has also been realized that these preferentially accumulate in the liver and kidney and to a lesser extent in spleen, lymph nodes and bone marrow (Agrawal et al., 1995; Iversen et al., 1995; Graham et al., 1998; Geary, 2009; Straarup et al., 2010). Liver, as a primary location of oligonucleotide accumulation has received a greater level of attention with some of the most promising AON trials taking advantage of this effect, as seen with Mipomersen (Hovingh et al., 2013). Liver accumulation has been attributed to the role of this organ in clearance by the reticulum endothelium system (RES). This results from the abundant presence of phagocytic Kupffer cells, together with the high blood flow received and, importantly, the existence of a fenestrated vasculature with an average 100–200 nm pore diameter between endothelial lining cells (Wisse et al., 2008). It should be noted that the pharmacokinetics of AONs are dependent on chemistry, with the most favorable properties relating to the presence of PS linkages and the polyanionic character of the molecules. Thus, AONs based on PNA and PMO when administered as “naked” formulations *in vivo*, are rapidly cleared from circulation while showing poorer tissue distribution (Dirin and Winkler, 2013).

Tumor tissue also shares some of the abovementioned features, specially regarding its specific microvasculature characteristics (*viz*. for solid tumors). Fenestrations of 100–700 nm have been found in some tumor vessels, which together with a poor lymphatic drainage give rise to the enhanced permeability and retention effect (EPR) (Jang et al., 2003), responsible for the accumulation of macromolecules or nanoparticles in tumors. Another effect to consider is the usually high interstitial fluid pressure (IFP) in tumors that obviates the normal rapid convective flow from blood to the tissue interstitium (due to osmotic and hydrostatic pressure differences). This effect is counterproductive in terms of drug accessibility to the tumor tissue, which then has to rely in slow diffusion processes. A dense structure of interstitial matrix and cells also mounts a final barrier to the diffusion process (Chauhan et al., 2011). Finally, the uneven leakiness of vessels found in tumors further contributes to a highly heterogeneous process of drug penetration. Another consideration is that the larger the tumor the bigger the regional differences within the tumor itself. This is illustrated by the presence of a necrotic core with an almost complete absence of blood flow, a seminecrotic region with poor blood flow within un-branched vessels, a stable region with branched vessels and good flow and an active angiogenic front where blood flow is variable and can be substantially higher than in surrounding host normal tissues (Jain, 2012).

These hindrances can result in AONs despite reaching tumor tissue, not being able to accumulate to a significant extent in the tumor tissue, with the additional drawback of distributing unevenly throughout the tissue (Plenat et al., 1995; Delong et al., 1997; Devi et al., 2005).

Certainly these delivery issues hamper a more effective translation of anti-cancer antisense oligonucleotides to the clinic.

## Perspectives on AON vectorization for cancer therapeutics

Given the wide tissue distribution properties of AONs and their preferential accumulation in organs other than tumor tissue, this can lead to the necessity of using high amounts of AONs in order to reach a meaningful biological effect, raising concerns due to presence of high AON concentrations in unspecific tissue/organs. In addition, although some level of localization to tumor tissue is attained due to the EPR effect, there can be a large heterogeneity in the targeting and distribution of AONs between tumors and within the same tumor. Not achieving a homogeneous and abundant distribution of AONs to the entire tumor can result in differential intracellular concentrations of the AON affecting functional efficiency and ultimately leading to some cells evading the anti-cancer action.

The development of nanocarriers for AON delivery could have a positive contribution in AON anti-cancer efficiency while minimizing toxicity, although their utility must be evaluated in a case-by-case basis. Nanoparticle systems will be also affected by inter and intra-tumor heterogeneity, where differences between tumor mass strongly influence the EPR and IFP effects. In fact, the EPR effect is more prevalent in tumors of 100 mm^3^ which limits its use when targeting small or unvascularized primary or secondary tumor (metastases) (Adiseshaiah et al., 2010). While AONs associated with nanoparticle systems can take greater advantage of the EPR effect, when “naked” administration is employed these will be affected to a wider extent by IFP similarly to small drugs Interestingly, this could mean that free AONs could have an advantage when dealing with a tumor with a less disturbed vascular architecture or when tumor vasculature normalization drugs are used (Juliano et al., 2009; Chauhan et al., 2012). This view can, however, be too simplistic as shown in a work dealing with imaging and modulation of AON microdistribution in solid tumor xenografts (Mocanu et al., 2007). It was seen that a drug-induced decrease in IFP was not accompanied by an expected improved distribution of the AON, in contrast to what has been reported for some small drugs. This was attributed to a strong association of the AON with regions of necrosis/hypoxia or due to the effect of the drug promoting neovascularization and the less permeable status of the newly formed vessels. Also, one could reason that the tumor matrix and the specific collagen content along with the status of other fibrilliary proteins could affect distribution of AONs (Netti et al., 2000; Mocanu et al., 2007), especially when dealing with PS-AONs due to their unspecific binding properties. In contrast to tumor normalization, the EPR effect can be transiently augmented by modulation of blood pressure and local increase of blood flow through the use of angiotensin-II-induced hypertension and nitric oxide releasing agents (Fang et al., 2011). In this way uptake of nanoparticle systems could be favored.

In terms of available systems for vectorization of AONs these can be divided in nanoparticle systems formed by interactions of different carrier formulations with the AONs or nanoconjugates where AONs are covalently linked to different functional molecules (e.g., peptides, sugars) (Juliano et al., 2012; Yin et al., 2014).

Carrier formulations that have been frequently used for delivery of different nucleic acids comprise cationic lipids and polymers. The basic driving force of complex formation is the electrostatic interaction. In brief, the carrier system needs to (i) protect the nucleic-acid from extracellular and intracellular degradation, until it reaches its target, (ii) achieve a prolonged circulation time in order to be accumulated in the location of interest, (iii) efficiently interact with the cellular membrane to promote uptake (generally through endocytosis processes), (iv) promote escape from endocytic vesicles and finally (v) dissociate from the active nucleic-acid in order for it to function (Yin et al., 2014).

Cationic lipids generally used with nucleic acids (forming lipoplexes) comprise DOTMA, DOSPE, DOTAP, but also neutral lipids such as the fusogenic DOPE have been incorporated to improve transfection efficiency (Simoes et al., 2005). Some of these lipids have been studied specifically with AONs (Jaaskelainen et al., 2000; Meidan et al., 2001; Gokhale et al., 2002) but few have been utilized in pre-clinical or clinical work. A liposome formulation of c-raf antisense oligonucleotide constitutes the first example of an AON-lipoplex taken into clinical development stages (Zhang et al., 2009).

Polymers have been also used. These have an immense chemical diversity and are easy to chemically manipulate thus enabling tuning of properties by functionalization. Some examples of polymeric systems that have been utilized are poly(L-Lysine) (Stewart et al., 1996) and poly(ethylene imine) (Seong et al., 2006). However, some issues regarding efficiency and toxicity have warranted the development of other systems based on natural and biodegradable polymers such as chitosan (Gomes et al., 2014).

Also worth mentioning are delivery systems based on inorganic nanoparticles, an emerging field, of which, gold nanoparticles are perhaps the most representative ones (Ding et al., 2014). A detailed view on the intracellular transport (e.g., understanding its endocytic route) (Wu et al., 2014) and careful evaluation of toxicity profile (e.g., genotoxicity, membrane damage) (Alkilany and Murphy, 2010) can provide important information to the advancement of the technology into clinical development.

Taking into consideration the previously mentioned tumor features, some design specificities should be taken into account when implementing an AON-nanocomplex strategy as anti-cancer therapeutic platform. Regarding size, the smaller the particle the better the intra-tumoral transport (<10 nm), however the EPR effect will be less significant than for bigger particles (10–200 nm) (Netti et al., 2000). On the other hand, particles on the higher size range will present a limited capacity to extravasate from vessel pores, but for the same reason will be more specific. Also, bigger sizes determine a higher clearance by the RES, although this can be counteracted by steric stabilization through poly(ethylene glycol) surface modification (Van Vlerken et al., 2007).

Surface charge also plays a crucial role. While cationic particles tend to target tumor endothelium and exhibit a higher vascular permeability than neutral or anionic ones, the fastest and more homogenously distribution in tumor interstitium is seen for the neutral particles. Presence of charge in particles contributes to aggregation with different components of the tumor matrix thus hindering transport. Accordingly, neutral or zwitterionic particles, or even particles with the property to change charge according to the microenvironment should perhaps be the best options (Chauhan et al., 2011). Shape, an often-overlooked property, likewise affects transport. Here factors such as rigidity and form (spherical vs. rod) come into play with flexible nanometer-sized particles showing, in principle, better transport characteristics (Chauhan et al., 2011).

In conclusion, the field of anti-cancer AONs is rapidly advancing, supported in part by the growing number of chemical modifications that conferred superior properties to AONs. However, specific and efficient delivery to tumors is still of uttermost importance. Uniform distribution throughout the tumor is an important challenge particularly due to intra-tumoral regional specificities and a progressive microenvironment. A further challenge lies in the dynamic nature of tumors that may correlate with temporal and spatial changes in expression of the AON target genes.

Multi-gene targeting AONs and efficient tumor targeting vectorization systems will, thus, be of uttermost importance in the development of a successful anti-cancer AON strategy.

### Conflict of interest statement

The authors declare that the research was conducted in the absence of any commercial or financial relationships that could be construed as a potential conflict of interest.
